# High attenuation value in non-contrast computer tomography can predict pyonephrosis in patients with upper urinary tract stones

**DOI:** 10.1097/MD.0000000000030557

**Published:** 2022-09-30

**Authors:** Xiaofei Lu, Dechao Hu, Benzheng Zhou

**Affiliations:** a Department of Urology, Xiang Yang No. 1 People’s Hospital Affiliated to Hubei University of Medicine, Xiangyang, China.

**Keywords:** Hounsfield unit, hydronephrosis, renal pyonephrosis, upper urinary tract stones, urogenic sepsis

## Abstract

To evaluate whether the higher attenuation value [Hounsfield unit (HU)] in non-contrast CT can predict pyonephrosis in patients with upper urinary tract stones (UTS). Between October 2019 and October 2021, patients with hydronephrosis or pyonephrosis secondary to upper UTS were retrospectively searched in our study. All patients with UTS were treated with percutaneous nephrostomy, percutaneous nephrolithotomy, retrograde ureteral stent or transurethral ureteroscope lithotripsy. We excluded patients treated with extracorporeal shock-wave lithotripsy. Patients whose CT was not performed in our hospital or treated in another hospital were also excluded. Clinical data regarding basic information, clinical feature, Calculi-related indicators, HU values of the renal pelvis, the thick wall of the renal pelvis on CT were collected. Univariate and multivariate logistic analyses were performed. Receiver operative characteristic curves were drawn to predict pyonephrosis. A total of 240 patients with UTS were retrospected in this research, 191 patients had hydronephrosis (Group 1), and 49 patients had hydronephrosis with pyonephrosis (Group 2). The HU value of the renal collecting system in Group 2 (mean, 15.46; range, +1/+30) was significantly higher than that in Group 1 (mean, 5.5; 5 range −6/+24) (*P* = .02); the receiver operative characteristic curve analysis revealed that the best cut-off value of 9.5 could predict the presence of pyonephrosis, with 71.4% sensitivity and 70.2% specificity (area under the curve = 0.613; 95% CI: 0.514–0.713). In this study, we found the HU attenuation value of the renal collecting system can be used to distinguish pyonephrosis from hydronephrosis in patients with UTS.

## 1. Introduction

Calculi-related obstructive pyonephrosis (COP) is a common disease occurring in about 3.2% of patients with upper urinary stones in urology.^[1]^ COP is considered a urological emergency, and it can cause a rapid loss of renal function and can quickly develop to urosepsis or even septic shock within hours.^[2]^ Studies have been published showing that urinary tract obstruction combined with infection significantly increases the risk of urosepsis and septic shock. The morbidity and mortality rate were high even after a emergency decompression.^[1–3]^ So early diagnosis and treatment of COP are particularly important to protect renal function and prevent septic shock.

In such cases, urine leukocyte and urine bacterial cultures usually do not accurately reflect the infection status in the kidney because of urinary tract obstruction. Several studies have shown that >50% of patients with pyonephrosis had negative urine culture results.^[4–6]^ Primary drainage, including percutaneous nephrostomy (PCN) or retrograde ureteral intubation, is the principal treatment for pyonephrosis. The gold standard diagnosis of pyonephrosis is pyogenic fluids during endoscopic surgery or primary drainage.^[2,7,8]^

So, some scholars have proposed that preoperative imaging examinations may help to determine the status of renal infection and diagnose pyonephrosis as soon as possible.^[8–14]^ CT is the most commonly used method for preoperative diagnosis of upper urinary calculi; it can quickly determine the location and size of stones in patients and the severity of hydronephrosis of the affected kidney. Some scholars have used CT attenuation values to predict positive urine culture, and they found it is high sensitivity and specificity.^[11]^ Boeri et al^[6]^ reported that Hounsfield unit (HU) attenuation could differentiate pyonephrosis from hydronephrosis. But few studies have examined the risk factors of calculi-associated obstructive pyonephrosis, and the development of pyonephrosis in COP is unfamiliar. Therefore, we performed a retrospective study to evaluate the prevalence and risk factors of COP and the role of CT attenuation values in predicting COP.

## 2. Materials and Methods

### 2.1. Patient’s collection

After ethical approval from the institutional ethical committee, the clinical data of patients with Calculi-related obstructive hydronephrosis in our hospital between October 2019 and October 2021 were retrospectively reviewed. A total of 240 patients admitted with upper urinary tract stones (UTS) were retrospected in this research. Patients without pyonephrosis in our research were admitted in Group 1 and patients with pyonephrosis were included in Group 2.

### 2.2. Inclusion and exclusion criteria

Inclusion criteria were: having complete clinical information; abdominal CT of patients must have been performed in our hospital, and all UTS patients must have been treated with either PCN, percutaneous nephrolithotomy, ureteral retrograde stent, or retrograde ureteroscope lithotripsy (URL).

The exclusion criteria were: patients without complete clinical information or abdominal CT not performed in our hospital; the patient who had been treated for calculi in another hospital before admission; patients treated with extracorporeal shock-wave lithotripsy or had bilateral UTS; and abnormal urinary tract anatomy.

### 2.3. Clinical data collection and criteria

The clinical data of patients were collected in 3 categories (patient-related, infection-related, and calculus-related). Patient-related data included the basic information about patients, and previous renal surgery details. The infection-related data included white blood cells (WBC), neutrophils, serum CRP, urine leukocyte, urine culture, and serum creatinine. The calculus-related data included number of stones, stone location, size and stone density, grade of hydronephrosis, duration of symptoms, HU values of the renal pelvis, and the thick wall of the renal pelvis.

Pyonephrosis was diagnosed by the presence of pyogenic fluids during endoscopic surgery or primary drainage (PCN and ureteral retrograde stent). Hydronephrosis was evaluated by ultrasound based on Onen Grading System.^[15]^

Grade 1 = renal pelvic dilation alone; Grade 2 = pelvic + caliceal dilation, renal parenchyma are normal; Grade 3 = pelvic + caliceal dilation, the medulla is short and thin, the cortex is normal; Grade 4 = pelvic + caliceal dilation, there is no medulla, the cortex is thin.

Two physicians calculated the HU values of the renal pelvis by using the free drawing region-of-interest method, and determined whether the wall of the renal pelvis was thickened. Stone size was measured on kidney ureter bladder; urine was taken for culture on the first day in the hospital for all patients; in Group 2, the pyogenic urine in the renal pelvis during surgery or emergency decompression was also taken for culture.

Urogenic sepsis was based on quick sepsis-related organ failure assessment score of 2 or higher.^[16]^

### 2.4. Statistical analysis

Student *t* test was performed in continuous data. Chi-Square test was used in categorical data, the clinical information of all patients was analyzed by univariate logistic analysis, we performed multivariate logistic regression analyses to the variables that were statistically significant in univariate analyses. All the risk factors were found, and the receiver operative characteristic curves that evaluated the accuracy were performed. *P* < .05 was significant.

## 3. Results

A total of 240 patients with UTS were retrospected in this research, 191 patients with hydronephrosis were admitted in Group 1, and 49 patients were admitted in Group 2 with pyonephrosis. There were no significant difference between the 2 groups in duration of symptom, associated comorbid condition, hematuria, and body mass index and age (*P* > .05). But analysis showed significant differences in sex (*P* = .01), fever (*P* = .001), and history of same-side urological surgery (*P* = .001) (Table 1).

**Table 1:** Data regarding patient-related factors.

	Patient with Hy	Patient with Py	*P* value
Patients (%)	191 (79.6)	49 (20.4)	
Age (yr)			.62
Mean ± SD	51.8 ± 12.16	56.82 ± 11.55	
Range	16–80	23–77	
Sex (male/female)	134/57	25/24	.01
Body mass index			.37
Mean ± SD	23.18 ± 3.78	22.65 ± 3.18	
Range	16.5–30.8	16.6–32.5	
Associated comorbid condition (n/N)			
DM	41/191	7/49	.26
Hypertension	74/191	14/49	.18
DM with HTN	13/191	3/49	.86
HBV	5/191	1/49	.81
HCV	1/191	0	
Tuberculosis	2/191	0	
Fever (n/N)	15/191	26/49	.001
Hematuresis (n/N)	73/191	13/49	.08
History of same-side urological surgery (n/N)	52/191	25/49	.001
Herpes labialis	0	26/49	

DM = diabetes mellitus, HBV = hepatitis B virus, HCV = hepatitis C virus, HTN = hypertension, Hy = hydronephrosis, Py = pyonephrosis, SD = standard deviation.

**Figure 1.**
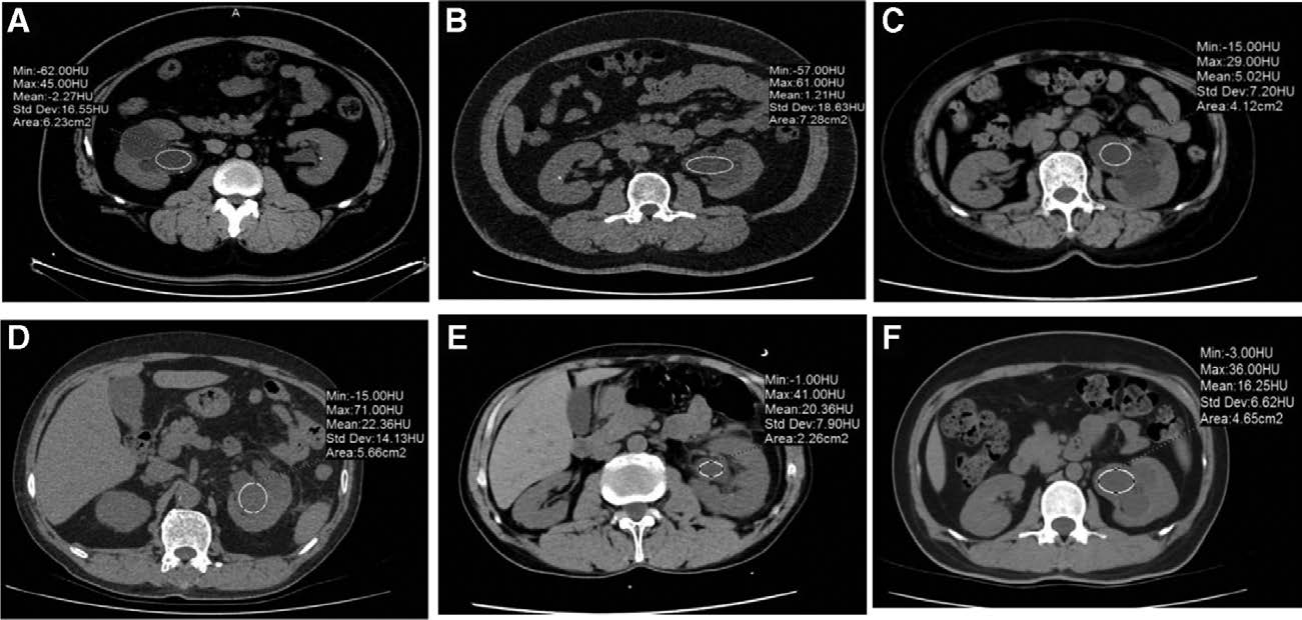
Hounsfeld units measurement of 3 different patients in pyonephrosis and Hydronephrosis group: (A–C) different patients in Hydronephrosis group; (D–F) different patients in pyonephrosis group.

In addition, we compared infection-related and calculus-related factors between the 2 groups. The HU value of renal collecting system in Group 2 was significant higher (*P* = .02), and analysis showed significant differences between the 2 groups in WBC count (WBCC), neutrophils, serum CRP (*P* < .001), serum albumin (*P* = .004), urine leukocyte (*P* = .029), WBCC (*P* < .001), urine culture in midstream urine (*P* < .001), grade of hydronephrosis (*P* = .02), and thick wall of the renal pelvis (*P* = .01) (Table 2 and Fig. 1).

**Table 2:** Data regarding infection-related and calculus-related factors.

	Patient with Hy	Patient with Py	*P* value
Patients (%)	191 (79.6)	49 (20.4)	
WBC (×10^9^/L)	7.63 (2.73–17.32)	12.97 (2.14–30)	<.001
Neutrophils (×10^9^/L)	5.30 (1.37–14.03)	11.02 (2.01–27)	<.001
Serum CRP (mg/L)	14.25 (0.28–252.12)	102.82 (0.77–309)	<.001
Serum albumin	42.15 (12.25–53.6)	34 (19.9–43.5)	.004
Urine leukocyte (/µL)	178.94 (0–9433)	461.98 (0–5320)	.029
WBCC	0.26 (0–4)	3.18 (0–43)	<.001
Urine culture			<.001
Positive	15 (8.4%)	18 (36.7%)	
Negative	164 (91.6%)	31 (63.3%)	
Location of stone (n/N)			
Ureteral calculus	161/191	45/49	.18
Renal calculus	100/191	23/49	.49
RC with UC	72/191	19/49	.89
Stone size (mm)	10.28 (6–28)	10.31 (6–27)	.38
Stone density (HU)	919.4 (150–1669)	793 (198–1559)	.23
Grade of hydronephrosis (n/N)			.02
Grade ½	94/191	15/49	
Grade ¾	97/191	34/49	
CT value of hydronephrosis (HU)	5.55 (-6–24)	15.46 (1–30)	.02
Staghorn calculi (n/N)	26/191	6/49	.8
Thick wall of the renal pelvis (n/N)	60/191	25/49	.01
Definitive surgery (n/N)			
PCNL	56/191	17/49	.46
URL	145/191	34/49	.62
PCNL and URL	10/191	2/49	.74

HU = Hounsfield Unit, Hy = hydronephrosis, PCNL = percutaneous nephrolithotomy, Py = pyonephrosis, RC = renal calculus, UC = ureteral calculus, URL = ureteroscope retrograde lithotripsy, WBC = white blood cells, WBCC = white blood cells count.

Then univariate and multivariate analyses of significant factors were performed for predicting pyonephrosis. The univariate analysis showed significant differences in sex, fever, history of urological surgery, WBCC, neutrophils, serum CRP, serum albumin, urine leukocyte, WBCC, grade of hydronephrosis, CT value of hydronephrosis, and thick wall of the renal pelvis (*P* < .05) between 2 groups. So, these 12 factors were admitted in the multivariate logistic analysis, then we found the history of urological surgery, WBCC, neutrophils, serum CRP, serum albumin, WBCC, grade of hydronephrosis, CT value of hydronephrosis were statistically significant for pyonephrosis (Table 3).

**Table 3:** Factors in univariate and multivariate analysis for predicting pyonephrosis before operation.

Variable	Univariate analysis		Multivariate analysis	
	OR (95% CI)	*P* value	OR (95% CI)	*P* value
Sex (male/female)	0.206 (0.105–0.404)	<.001	0.522 (0.098–2.775)	.446
Diabetes mellitus	1.122 (0.793–1.587)	.262		
Hypertension	0.938 (0.647–1.359)	.184		
Duration of symptom (d)	1.193 (0.613–2.322)	.293		
Fever (n/N)	10.020 (4.936–20.340)	<.001	2.447 (0.332–18.004)	.380
Hematuresis (n/N)	0.453 (0.213–0.964)	.078		
History of urological surgery	6.292 (3.152–12.559)	.001	5.786 (1.041–32.148)	.045
WBC (×10^9^/L)	0.116 (0.058–0.232)	<.001	0.074 (0.011–0.480)	.006
Serum CRP (mg/L)	0.035 (0.008–0.149)	<.001	0.018 (0.001–0.329)	.007
Serum albumin	0.039 (0.015–0.105)	.004	0.128 (0.018–0.902)	.039
Urine leukocyte (/µL)	0.213 (0.080–0.564)	.002	5.915 (0.535–65.407)	.147
WBCC	0.100 (0.042–0.237)	<.001	0.052 (0.004–0.624)	.020
Urine culture	0.132 (0.059–0.298)	<.001	1.063 (0.131–8.618)	.954
Stone density (HU)	0.550 (0.281–1.076)	.233		
Grade of hydronephrosis (n/N)	0.388 (0.204–0.738)	.021	0.134 (0.024–0.733)	.020
CT value of hydronephrosis (HU)	0.019 (0.006–0.055)	<.001	0.050 (0.007–0.343)	.002
Thick wall of the renal pelvis	4.810 (2.457–9.416)	.012	4.867 (0.805–29.411)	.085

CI = confidence interval, HU = Hounsfield Unit, Hy = hydronephrosis, OR = odds ratio, WBC = white blood cells; WBCC = white blood cells count.

**Figure 2.**
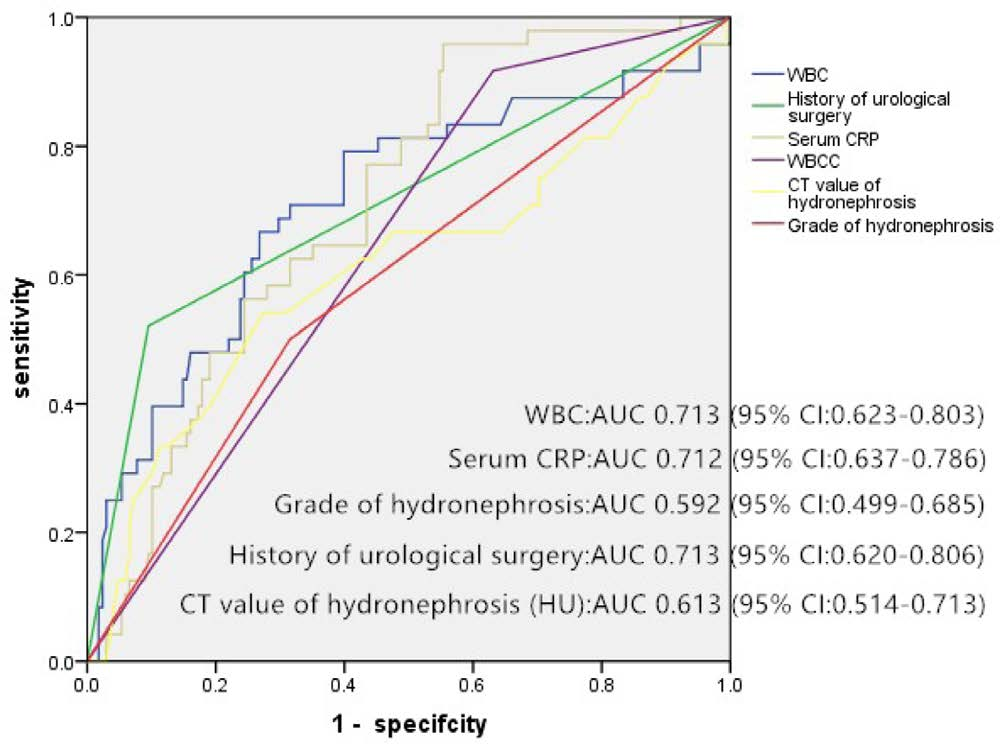
The sensitivity and specificity of independent risk factors in predicting pyonephrosis in patients with upper urinary tract stones in ROC analysis. ROC = receiver operative characteristic.

Receiver operative characteristic analysis revealed that WBCC (area under the curve [AUC] = 0.713), history of urological surgery (AUC = 0.713), serum CRP (AUC = 0.712), and HU value of hydronephrosis (AUC = 0.613) had a good ability to predict pyonephrosis in patients with UTS (Fig. 2).The sensitivity and specificity of the HU value in predicting COP were 71.4% and 70.2%, respectively; the optimal cut-off value was 9.5.

## 4. Discussion

UTS are among the most common diseases in urology, which often cause hydronephrosis or even renal pyonephrosis. And, COP occurs in about 3.2% of patients with UTS.^[1]^ COP is considered a urological emergency, and it can cause a rapid loss of renal function and can quickly develop to urosepsis or even septic shock within hours.^[2]^ In recent years, the incidence of sepsis caused by UTS has increased, and the associated mortality rate is still high, although the management of the patients has improved.^[2,3]^ So early diagnosis and treatment of COP are particularly important to protect renal function and prevent septic shock.

The evolution of renal pyonephrosis has not been exactly investigated. Obstruction caused by UTS and bacterial infection are 2 primary pathogenesis of COP.^[4,5]^The severity of hydronephrosis depends on the ureteral obstruction, in this study, higher degree of hydronephrosis was found in patients with renal pyonephrosis than those without pyonephrosis. And, the multivariate logistic analysis showed that the grade of hydronephrosis is an independent factor of COP. Boeri et al^[6]^ also found that the grade of hydronephrosis is an independent predictor of COP, and they think patients with higher grade of hydronephrosis (grades III–IV) are 3 times more at risk of renal pyonephrosis than patients with lower grade of hydronephrosis (grades I–II). Hydronephrosis increases pressure in the renal pelvis and reduces urine formation, so the risk of retrograde infection by bacteria is significantly increased.

*Escherichia coli* is the most common infecting organism in urinary tract infection (UTI) due to retrograde infections.^[6,17,18]^ In our study, *E. coli* was also the most common infecting organism (32.5% of cases). So, the preoperative urine leukocyte count and WBBC were higher in patients with pyonephrosis than in the hydronephrosis group. But the positive rate of mid-stream urine culture (MUC) was only 36.4% in patients with pyonephrosis. In our study, 33.3% of patients’ renal pelvis urine cultures were positive, and preoperative mid-stream urine cultures were negative in patients with pyonephrosis. Patodia et al^[7]^ were also got the similar results, they found the positive rate of midstream urine culture below 50% in patients with pyonephrosis. Liu et al^[19]^ also found preoperative urine culture played a role in predicting SIRS after percutaneous nephrolithotomy, but it was unable to prevent the occurrence of SIRS. Because of the urinary obstruction, the infection may persist in the upper system when the MUC is negative. Although far higher than that in patients with hydronephrosis, we believe the MUC cannot be a good predictor of COP because of the low positive rate.

Previous studies have observed that women are more likely to suffer from UTIs than men, but males are more likely to suffer from urolithiasis.^[4,19,20]^ In this study, 24 of 49 patients with pyonephrosis are women. Therefore, we propose that female patients with UTS are more likely to suffer from renal pyonephrosis because of the high risk of UTI. In addition, 15 of 24 female patients with pyonephrosis developed urogenic sepsis and only 3 males developed urogenic sepsis. We believe that the difference between genders is significant.

Primary drainage, including PCN or retrograde ureteral intubation, is the principle for diagnosing and treating pyonephrosis.^[7,8,21]^ And, whether the preoperative imaging examinations can be used to predict the presence of pyonephrosis in patients with UTS.

Radiological examinations, as ultrasound, MRI, and CT, are important in the diagnosis of pyonephrosis because they can achieve a rapid result and help determine the status of renal infection. Ultrasound is a conventional diagnostic method for upper urinary calculi. It has been reported that renal pyonephrosis may be suggested when strong echo points, uneven density, and blurred images occur in the renal collecting system.^[8]^ However, ultrasound is highly subjective and has no quantitative indicators, limiting the value of renal pyonephrosis diagnosis. Magnetic resonance (MR) is a reliable tool to differentiate pyonephrosis from hydronephrosis but is not widely used in the diagnosis of upper urinary calculi due to its low efficiency in diagnosing calculi.^[9,10]^ CT is by far the most commonly used method for diagnosing upper urinary calculi, and the diagnostic rate of calculi is as high as 95%–100%. Meanwhile, it can clearly show the renal parenchyma, renal collecting system, and perirenal conditions.^[11]^

The HU is the scale of tissue density at standard temperature and pressure, related to 2 predefined values: air defined as −1000 HU and water 0 HU.^[22]^ Physicians routinely use the HU attenuation value measured by computed tomography to assess the hardness of urinary calculi, helping to determine the stone treatment and define the nature of the kidney masses.^[12,13,23,24]^

Stunell et al^[14]^ found non-specific features in computed tomography in patients with pyelonephritis. Also, in their study, they did not measure the HU value of the renal collecting system. However, Yuruk et al^[25]^ found that the HU value of the renal collecting system in patients with pyonephrosis was significantly higher than in patients with hydronephrosis in a retrospective study of 105 patients with calculi-related obstructive hydronephrosis. The best cut-off value for predicting pyonephrosis was 9.21 (65.9% sensitivity and 87. 9% specificity). We also report similar results; HU values of the dilated renal collecting system of patients with pyonephrosis were higher than patients with hydronephrosis (*P* = .02). The sensitivity and specificity of the HU value in predicting COP were 71.4% and 70.2%, respectively; the optimal cut-off value was 9.5.

## 5. Limitation of study

A limited number of patients did not have the data for calculus component analysis, so we were unable to test the relationship between calculus components and pyonephrosis. There was no clear boundary between infective hydronephrosis and pyonephrosis, and there were certain deviations in the collected data. A large-scale case analysis is now needed to continuously confirm the conclusions.

## 6. Conclusions

In this study, we found that many factors, such as the history of urological surgery, lower serum albumin, and grade of hydronephrosis, are associated with the development of COP. The HU attenuation value of the renal collecting system can distinguish pyonephrosis from hydronephrosis in patients with UTS.

## Author contributions

Conceptualization: Xiaofei Lu, Dechao Hu, Benzheng Zhou.

Data curation: Dechao Hu.

Formal analysis: Dechao Hu.

Visualization: Xiaofei Lu.

Writing – original draft: Xiaofei Lu, Benzheng Zhou.

Writing – review & editing: Xiaofei Lu, Dechao Hu, Benzheng Zhou.
